# Pregnancy Inhibits Mammary Carcinogenesis by Persistently Altering the Hypothalamic–Pituitary Axis

**DOI:** 10.3390/cancers13133207

**Published:** 2021-06-26

**Authors:** Ramadevi Subramani, Adriana Estrada, Madeline Dixon, Maria Parada, Sheryl Rodriguez, Diego A. Pedroza, Matthew D. Ramirez, Alexa Clift, Lilia Garcia, Rajkumar Lakshmanaswamy

**Affiliations:** 1 Center of Emphasis in Cancer, Department of Molecular and Translational Medicine, Paul L Foster School of Medicine, Texas Tech University Health Sciences Center El Paso, El Paso, TX 79905, USA; ramadevi.subramani@ttuhsc.edu (R.S.); sheryl.rodriguez@ttuhsc.edu (S.R.); 2 Graduate School of Biomedical Sciences, Texas Tech University Health Sciences Center El Paso, El Paso, TX 79905, USA; maria.parada@ttuhsc.edu (M.P.); diego.a.pedroza@ttuhsc.edu (D.A.P.); alexa.clift@ttuhsc.edu (A.C.); lilia.garcia@ttuhsc.edu (L.G.); 3 Department of Cell Biology & Biochemistry, Texas Tech University Health Sciences Center, Lubbock, TX 79430, USA; adriana.estrada@ttuhsc.edu; 4 Paul L Foster School of Medicine, Texas Tech University Health Sciences Center El Paso, El Paso, TX 79905 USA; madeline.dixon@ttuhsc.edu (M.D.); matthew.d.ramirez@ttuhsc.edu (M.D.R.)

**Keywords:** pregnancy, mammary cancer, hypothalamus, pituitary, hormones

## Abstract

**Simple Summary:**

Breast cancer is one of the most frequently diagnosed cancers and it is the second leading cause of cancer-related death in women. Early first full-term pregnancy has been known to reduce the life-time risk of breast cancer. The actual mechanism by which pregnancy reduces the life-time risk of breast cancer is not well understood. It is well established that hormones are vital for a successful full-term pregnancy and they can also influence the risk of breast cancer. The emphasis has been placed mainly on the ovarian hormones estrogen and progesterone. It is also known that hypothalamic and pituitary hormones can impact the breast. In this study, we investigated how pregnancy alters the hypothalamic/pituitary hormones and what effect these hormonal alterations have on the risk of breast cancer development. Our results demonstrate that pregnancy persistently alters the hypothalamic–pituitary hormonal axis leading to the reduction of breast cancer risk.

**Abstract:**

Early full-term pregnancy is known to reduce the lifetime risk of breast cancer. Although the phenomenon of parity-induced protection is well-established, the physiological mechanisms involved in this protection are not clear. Earlier reports have shown that pregnancy results in alterations of hormone levels. How pregnancy affects hypothalamic hormones and how the change, if any, influences breast cancer is not well understood. Seven-week-old female Lewis rats were given N-methyl-N-nitrosourea. Two weeks post carcinogen exposure, a set of females were housed with males to generate the parous rats and another set of rats served as the nulliparous controls. Mammary tumorigenesis was assessed for 9 months. Hypothalamic and pituitary levels of hormones were measured at various timepoints. Further, animals were also challenged with growth hormone and prolactin secretagogues to test the effect of pregnancy on the hypothalamic–pituitary hormonal axis. Persistent alterations in the level of growth hormone-releasing hormone, thyrotropin releasing hormone, dopamine, and somatostatin in the hypothalamus of parous animals was observed. Further, we also observed that pregnancy had a significant effect on the pituitary gland and its response to growth hormone and prolactin secretagogues. Our studies using the rodent model system demonstrate that pregnancy could be reducing the risk of breast cancer by persistently altering the hypothalamic–pituitary axis, which could have implications for breast cancers in humans as well.

## 1. Introduction

A full-term pregnancy before the age of 20 years is the only known natural phenomenon that can drastically reduce the risk of breast cancer in women of all ethnic backgrounds worldwide [1,2,3,4,5,6]. This universal protective effect of early pregnancy is a major consideration in developing preventive strategies against breast cancer. It has also long been known that parous rats and mice, in contrast to their nulliparous counterparts, develop fewer, if any, mammary tumors after the administration of carcinogens [7,8,9]. Furthermore, the protective effects of pregnancy have been reported in rats undergoing pregnancy before or after exposure to chemical carcinogens. Commonly used mammary carcinogens are N-methyl-N-nitrosourea (MNU) and dimethylbenzanthracene. These carcinogens cause alterations in DNA structure and lead to the development of mammary cancers, which are mainly estrogen receptor positive.

It has been suggested that this protective effect is likely due to differentiation of the target structures during carcinogenesis, involving the terminal end buds and terminal ducts, by hormones associated with pregnancy [10,11,12]. Based on comparisons between parous and nulliparous women, it has also been shown that parous women have reduced circulatory levels of prolactin and androgens, increased estriol, and elevated levels of sex hormone binding globulins. These systemic changes are also thought to be associated with the protective effects of pregnancy. Additionally, there is a significant decrease in the circulating levels of growth hormone and prolactin, and decreased levels of estrogen and epidermal growth factor receptors in mammary glands of parous rats, when compared to age-matched nulliparous (AMNP) rats [7]. Finally, changes in mammary stem cell characteristics in terms of self-renewal, morphogenesis, and signaling capabilities have also been suggested as a reason for early parity-induced protection against breast cancer [13,14,15,16].

Pregnancy-induced neuroendocrine changes and how they affect breast cancer are not well understood. Earlier reports have shown that pregnancy results in long-term persistent alterations in pituitary hormones, many of which are controlled by hypothalamic hormones. How pregnancy affects hypothalamic hormones and how the change, if any, influences breast cancer is not well understood. The two main pituitary hormones that are persistently altered are growth hormone (GH) and prolactin (PRL). These two hormones are directly under the control of hypothalamic factors. GH is synthesized and secreted in response to growth hormone-releasing hormone (GHRH) and is inhibited by somatostatin [17,18,19], while PRL is synthesized in response to thyrotropin-releasing hormone (TRH) and is inhibited by dopamine [20,21,22]. These hypothalamic factors could play crucial roles in breast cancer prevention.

Although the phenomenon of parity-induced protection against mammary carcinogenesis is well established, the physiological mechanisms involved in this protection are not well understood. The development of novel preventive strategies to reduce the risk of breast cancer without having to undergo pregnancy early in life is critical in understanding the underlying mechanism of this protective effect of pregnancy against breast cancer. In this report, we show that pregnancy reduces the risk of breast cancer by persistently altering the neuroendocrine regulation of hypothalamic and pituitary factors. These findings in the rodent model system might further highlight a potential physiological mechanism by which pregnancy reduces the risk of developing breast cancer in humans. 

## 2. Materials and Methods

### 2.1. Animals

All animal studies were approved by the Institutional Animal Care and Use Committee of the Texas Tech University Health Sciences Center (El Paso, TX, USA). We used 7-week-old female and male Lewis rats purchased from Envigo (Indianapolis, IN, USA). Female and male rats were housed in breeding cages. The male rats were isolated once the vaginal plug was observed. All parous rats were uniparous and had undergone 6 weeks of post-partum regression. We previously observed that by 4–6 weeks of age, the mammary gland undergoes optimal regression following differentiation induced by pregnancy, resulting in a mammary gland composed mainly of ductal structures with very few persisting lobules. The following line diagram shows the general experimental protocol as well as the experimental time points at which the samples were collected.

### 2.2. Experimental Design Line Diagram

**Figure 1:**
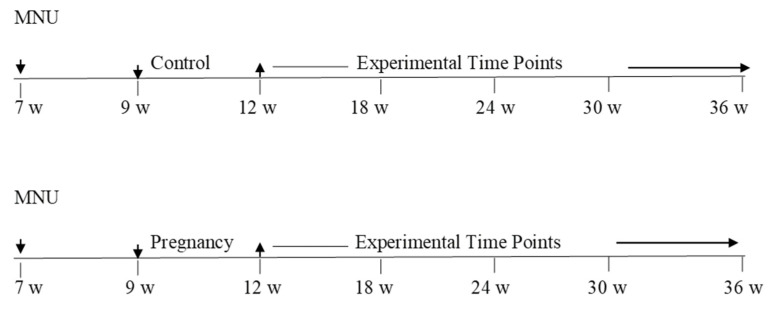
The line diagram of the respresentation of the experimental design.

The following line diagram is the respresentation of the experimental design that was used to conduct the experiments. Mammary carcinogenesis was followed upto 36 weeks. Blood and tissue samples were collected at each time and used for analysis (Figure 1).

### 2.3. Mammary Carcinogenesis

N-methyl-N-nitrosourea (MNU) was dissolved in 0.9% NaCl (pH 4.0–5.0) and injected intraperitoneally at a dose of 50 mg/kg body weight. All experimental animals were palpated twice weekly for the presence of mammary tumors. The palpable tumors were measured in two axes and were surgically removed under isoflurane anesthesia before they reached a size of 2 cm in any axis. Post-surgery the animals were administered rimadyl as analgesic for the following five days. The cancerous nature of the palpable tumors was confirmed by histopathological analyses.

### 2.4. Immunohistochemistry

Formalin-fixed paraffin-embedded mammary tumor tissues were sectioned using a microtome at 5 micron thickness. Tissue sections were incubated in an oven at 58 °C for 2 h, and then were deparaffinized using a xylene bath for 20 min. The sections were rehydrated in serial alcohol baths and were then placed in a distilled water wash for 5 min. Antigen retrieval was conducted using trilogy (Cell Marque, Rocklin, CA, USA) followed by blocking in tris-buffered saline (TBS) containing 1% fetal calf serum and 1% bovine serum albumin for 15 min. This was followed by the addition of peroxide-free blocking reagent (Cell Marque) for 10 min. Tissues sections were then incubated with various primary antibodies (1∶50–1:1000 dilution) overnight at 4 °C. The slides were then washed in phosphate-buffered saline (PBS) for 5 min (3×) and incubated with Ultra Marque polyscan HRP Label (Cell Marque) for 1 h at room temperature. Tissue sections were then washed in PBS and stained with 3,3’-diaminobenzidine chromogen (Cell Marque) for 20 min. Counterstaining was conducted with hematoxylin for 40 s. Tissue sections were then rinsed with distilled water and dehydrated with serial ethanol solutions, and then placed in a xylene bath. Finally, mounting medium (Surgipath Medical Industries, Richmond, IL, USA) was used to place a coverslip over the tissue sections. Images of stained tissues were captured using an Eclipse 50i microscope (Nikon, Tokyo, Japan).

### 2.5. Measurement of Static Levels of Hypothalamic and Pituitary Hormones in Response to Pregnancy

Parous rats were generated as described above. AMNP rats were used as controls. The animals were sacrificed at 18, 24, 30, and 36 weeks of age, and the hypothalamus and pituitary were removed and snap-frozen in liquid nitrogen for later extraction. We measured the content of GHRH and TRH to assess the effects of parity on GH- and PRL-stimulating peptides, respectively. GHRH is the main hypothalamic stimulator of GH secretion and TRH is the main hypothalamic stimulator of PRL from the pituitary gland. The content of the GH inhibitor, somatostatin (SS), and the PRL inhibitor, dopamine (DA), were also measured to determine whether parity affected their levels in the hypothalamus. Pituitary levels of GH, PRL, GHRH, and TRH were also determined. For the measurement of GHRH, we homogenized tissues in 2 M acetic acid. The homogenate was then boiled and lyophilized. The lyophilized material was reconstituted, extracted, and assayed using an enzyme immunoassay (EIA) kit (MyBioSource) following the manufacturer’s protocol. For measuring TRH, tissues were homogenized in PBS and extracted in methanol. After centrifugation, the supernatant was air-dried overnight and then reconstituted in PBS containing 0.25% bovine serum albumin and assayed using an EIA kit. For SS extraction, tissues were homogenized in PBS and SS was extracted in 0.5% trifluoroacetic acid (TFA). The supernatant was eluted with 60% acetonitrile and 1% TFA in water. The eluent was evaporated to dryness and the residual material was reconstituted in assay buffer and assayed using an EIA kit. DA was extracted and assayed according to the instructions for an EIA kit. The samples were determined in triplicate in all assays, and the results are expressed as the concentration per g of wet weight tissue. 

### 2.6. The Hypothalamic–Pituitary Axis Response to GH and PRL Secretagogues 

We determined whether an early parity resulted in a persistently altered response of the hypothalamic–pituitary axis to GH and PRL secretagogues. Perphenazine (PPZ), a dopamine receptor inhibitor that causes the acute release of PRL from the anterior pituitary by blocking the inhibitory effect of dopamine from the hypothalamus was used as the PRL secretagogue, and growth hormone-releasing peptide 6 (GHRP-6), a synthetic hexapeptide that causes the release of GH, was also used. We used these secretagogues to test the responsiveness of the hypothalamic–pituitary axis in parous and AMNP rats. The animals were generated as described in the line diagram shown above. The stage of the estrous cycle was determined by examination of vaginal cytology in groups of 10 rats at 18, 24, 30, and 36 weeks of age. The animals were anesthetized with isoflurane on the morning of diestrus for GH secretion studies. We anesthetized the animals with CO_2_ inhalation on the morning of proestrus for the PRL studies because isoflurane is known to significantly alter the levels of PRL. Three blood samples were drawn 5 min apart prior to the administration of the secretagogue to determine the basal serum concentrations for GH and PRL, and the experimental animals were then intravenously administered with three doses of GHRP-6 (100 ng, 500 ng, and 1 μg) or PPZ (10 ng, 100 ng, or 1 μg). Blood samples were then collected at 5, 10, and 15 min after injection of the secretagogues. In all cases, 0.25 mL of blood was collected. Serum was harvested and assayed for levels of GH and PRL using EIA kits.

### 2.7. Measuring the Functional Activity of the Pituitary in Response to GH and PRL Secretagogues

To determine if parity reduced the sensitivity of the pituitary to secretagogues, we conducted perfusion studies on isolated pituitaries. Experimental groups of rats were generated as described in the line diagram. The animals were sacrificed at the same states of the estrous cycle as described above for each test and the pituitaries were removed and washed in a serum-free medium. The pituitaries were then placed in perfusion micro chambers (Endotronics, Lisle, IL, USA) for experimentation. This perfusion system facilitated simultaneous testing of six samples under heat-controlled and CO_2_-buffering conditions, as well as with a wide range of flow rate settings. Medium 199 containing penicillin/streptomycin antibiotics was used for the perfusion. The pituitaries were perfused at a flow rate of 10–30 mL/h for 45 min prior to establishing a steady basal release of the hormones. Then, 5 min fractions were collected for 30 min and the secretagogues were administered. The samples were then collected at 5, 10, and 15 min post-secretagogue treatment and were assayed for GH and PRL using EIA kits.

## 3. Results

### 3.1. Parity Inhibits Mammary Carcinogenesis

**Figure 2:**
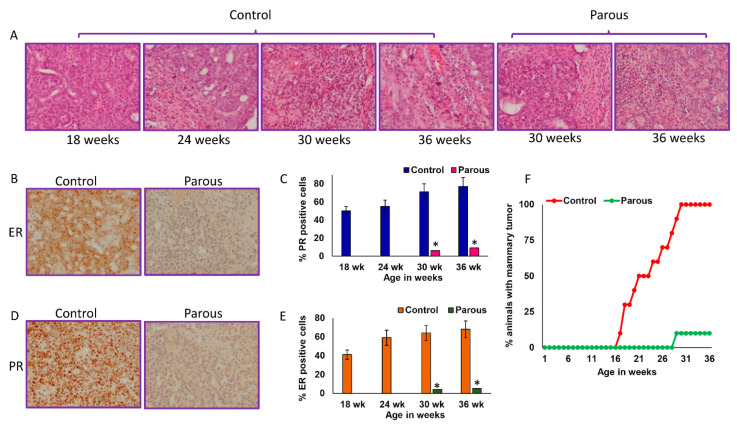
(**A**) Histology of mammary tumors from nulliparous control animals and parous animals at 18, 24, 30, and 36 weeks of age. (**B**) Representative picture of the estrogen receptor immunohistochemistry from nulliparous and parous mammary tumors. (**C**) Percent positivity for estrogen receptor in mammary tumors from nulliparous and parous animals at different timepoints. (**D**) Representative picture of the progesterone receptor immunohistochemistry from nulliparous and parous mammary tumors. (**E**) Percent positivity for progesterone receptor in mammary tumors from nulliparous and parous animals at different timepoints. (**F**) Mammary carcinogenesis observed over 36 weeks. * represents *p* < 0.05.

As expected, parous rats had a significantly lower incidence of palpable mammary tumors compared to age-matched nulliparous controls at all time points. At 9 months of age, only 10% of parous rats had mammary tumors (Table 1(A)). These data showed the protective effect of parity against mammary carcinogenesis. We also observed that age-matched nulliparous rats developed an average of 5 ± 1 mammary tumors per tumor-bearing rat. In contrast, parous rats that developed mammary tumors had only one tumor per rat (Table 1(B)). This further indicated that parity not only decreased the incidence of mammary tumors, but also inhibited mammary tumor incidence. The mammary tumors were histologically similar between the parous and nulliparous groups (Figure 2A). Nulliparous animals mainly developed ER+ and PR+ mammary tumors, while the few mammary tumors that developed in parous rats were ER−, PR−, or weakly positive (Figure 2B–E). Parous rats did not develop any mammary tumors until 29 weeks of age, while 30% of age-matched nulliparous rats developed mammary tumors by 18 weeks. By week 30, all age-matched nulliparous rats developed mammary tumors (Figure 2F). The average mammary cancer latency was significantly delayed in the parous rats (221 ± 25 day) compared to the nulliparous group (158 ± 32) (Table 1(C)).

### 3.2. The Effect of Parity on Tissue Levels of GHRH, TRH, DA, SS, GH, and PRL

**Figure 3:**
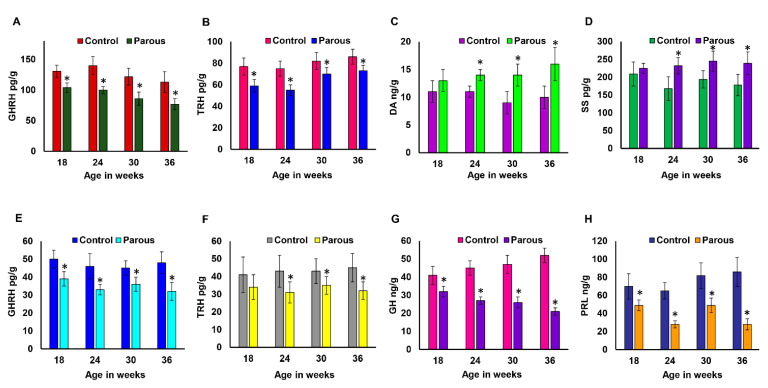
Pregnancy induces a persistent and significant change in the level of hormones in the hypothalamus and pituitary gland. All hormone levels were measured at 18, 24, 30, and 36 weeks old female parous and nulliparous Lewis rats (n = 10/group/timepoint). Hypothalamic levels of (**A**) growth hormone-releasing hormone (GHRH). (**B**) Thyrotropin releasing hormone (TRH). (**C**) Dopamine (DA). (**D**) Somatostatin (SS). Pituitary levels of (**E**) GHRH, (**F**) TRH, (**G**) growth hormone (GH), and (**H**) Prolactin (PRL). * represents *p* < 0.05.

We measured the levels of GHRH, TRH, DA, and SS in hypothalamic tissues of parous and AMNP rats at different time points. The hypothalamic levels of GHRH (Figure 3A) and TRH (Figure 3B) were significantly decreased in the parous group compared to the AMNP group at all time points. In contrast, the hypothalamic levels of DA (Figure 3C) and SS (Figure 3D) were increased in parous rats compared to AMNP rats. Our results indicated that parity significantly lowered the hypothalamic levels of GHRH and TRH, while the levels of DA and SS were higher in the parous hypothalamus group than in the AMNP group. We also measured the levels of GHRH and TRH in pituitary tissues. Both GHRH (Figure 3E) and TRH (Figure 3F) levels were consistently reduced in the parous group at all time points. Because GHRH and TRH are the major hormones responsible for the synthesis and secretion of GH and PRL, respectively, we also measured the pituitary levels of GH and PRL in AMNP and parous rats. We found that GH levels decreased with age in both groups. The levels of GH gradually decreased over time in the AMNP rats, while parous rats had a remarkable decrease in GH levels starting at 6 weeks post-weaning, which was also maintained at other time points (Figure 3G). However, prolactin levels increased with age in AMNP rats, with the highest levels found in older rats. The levels of prolactin in parous rats were not drastically different from the AMNP group at 6 weeks post-weaning, but the levels began to significantly decline at all later time points (Figure 3H). Together, these results showed that parity had a lasting effect on the neuroendocrine regulation of the hypothalamus and pituitary axes.

### 3.3. The Effect of Parity on the Hypothalamic–Pituitary–GH Axis

**Figure 4:**
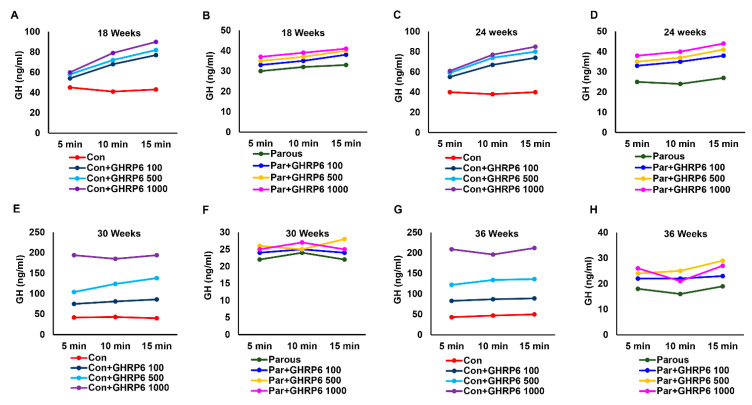
Circulating levels of growth hormone (GH) after administration of growth hormone-releasing peptide 6 (GHRP-6) at different doses to age-matched nulliparous and parous rats at different timepoints. (**A**,**C**,**E**,**G**) GH levels in age-matched nulliparous rats administered with GHRP6 (100, 500, 1000 ng) at 18, 24, 30, and 36 weeks. (**B**,**D**,**F**,**H**) GH levels in parous rats administered with GHRP6 (100, 500, 1000 ng) at 18, 24, 30, and 36 weeks.

Because we already estimated the tissue levels of GH and its regulators, we next determined the levels of circulating GH. The levels of GH (Figure S1) were significantly reduced in parous rats at all time points when compared to AMNP rats. This finding is significant because GH is known to promote breast cancer growth, and a lower circulating level of GH could be one of the reasons for the reduced risk of breast cancer in the parous group. We then determined if the response to stimuli of the hypothalamic–pituitary axis in parous rats was altered. This was tested by injection of various intravenous doses of GHRP-6, a GH secretagogue, to the AMNP and parous groups of animals at different time points. Administration of GHRP-6 resulted in a significant dose-dependent increase in GH levels in AMNP rats at all time points (Figure 4A,C,E,G). However, the increase in the levels of GH in response to GHRP-6 was significantly decreased in parous rats (Figure 4B,D,F,H). Together, the results indicated that the hypothalamic–pituitary–GH axis in parous animals had a decreased response to stimuli, when compared to AMNP rats.

### 3.4. The Influence of Parity on the Hypothalamic–Pituitary-PRL Axis

**Figure 5:**
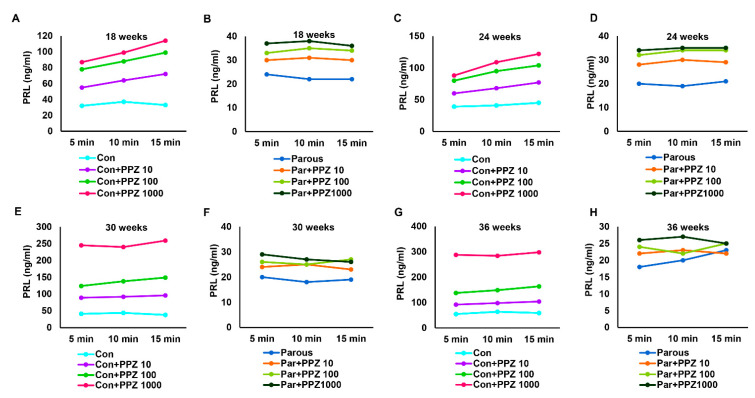
Circulating levels of prolactin (PRL) after administration of perphenazine (PPZ) at different doses to age-matched nulliparous and parous rats at different timepoints. (**A**,**C**,**E**,**G**) PRL levels in age-matched nulliparous rats administered with PPZ (10, 100, 1000 ng) at 18, 24, 30, and 36 weeks. (**B**,**D**,**F**,**H**) PRL levels in parous rats administered with GHRP6 (10, 100, 1000 ng) at 18, 24, 30, and 36 weeks.

The hypothalamic and pituitary levels of PRL and its regulators were determined and found to be altered in parous rats, so we measured the levels of PRL in the circulation at different time points. The results showed that PRL levels were not significantly altered in AMNP versus parous animals at 18 weeks of age, while its levels were significantly reduced in parous animals at all other time points when compared to AMNP animals (Figure S2). Like GH, PRL is also a known mammogenic hormone and influences mammary carcinogenesis. Lower circulating levels of PRL along with reduced GH could contribute to the reduced risk of breast cancer in parous animals. Similar to GH, we also measured the responses to stimuli activity of the hypothalamic–pituitary–PRL axis in parous rats using PPZ, a PRL secretagogue. Different doses of PPZ were intravenously injected into the AMNP and parous groups of animals at different time points, resulting in a significant dose-dependent increase in PRL levels in AMNP animals (Figure 5A,C,E,G). The responses of parous animals to PPZ were significantly decreased when compared to AMNP animals at all time points, and for all doses of PPZ (Figure 5B,D,F,G). Together, these results further showed that parity influenced the hypothalamic–pituitary–PRL axis.

### 3.5. Parity Induces Dynamic Changes in Pituitary Glands

**Figure 6:**
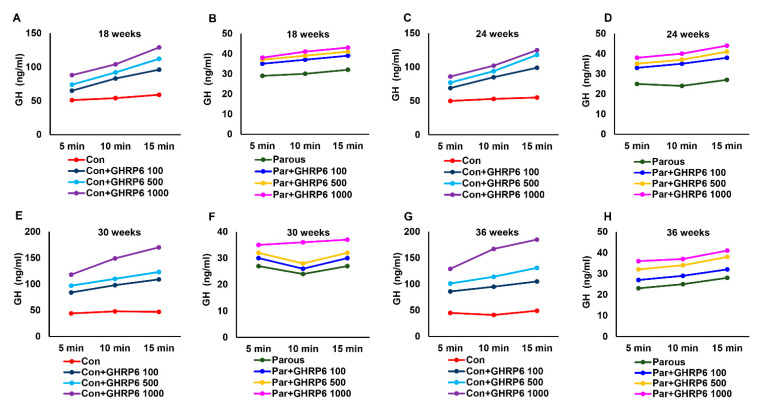
Amount of growth hormone (GH) secreted from the pituitary gland after treatment with growth hormone-releasing peptide 6 (GHRP-6) at different doses to age-matched nulliparous and parous rats at different timepoints. (**A**,**C**,**E**,**G**) GH levels in age-matched nulliparous rats treated with GHRP6 (100, 500, 1000 µg) at 18, 24, 30, and 36 weeks. (**B**,**D**,**F**,**H**) GH levels in parous rats treated with GHRP6 (100, 500, 1000 µg) at 18, 24, 30, and 36 weeks.

**Figure 7:**
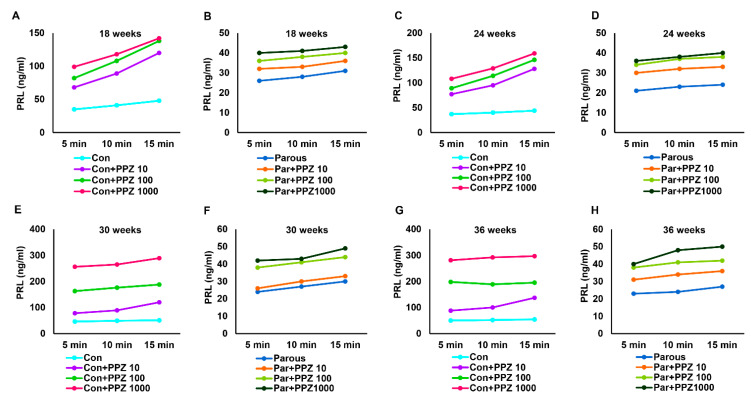
Amount of prolactin (PRL) secreted from the pituitary gland after treatment with perphenazine (GHRP-6) at different doses to age-matched nulliparous and parous rats at different timepoints. (**A**,**C**,**E**,**G**) GH levels in age-matched nulliparous rats treated with PPZ (10, 100, 1000 ng) at 18, 24, 30, and 36 weeks. (**B**,**D**,**F**,**H**) GH levels in parous rats treated with PPZ (10, 100, 1000 ng) at 18, 24, 30, and 36 weeks.

To further identify and differentiate the influences of parity on the hypothalamic–pituitary axis, pituitaries were isolated from parous and AMNP rats sacrificed at various time points. They were placed in individual temperature-controlled perfusion chambers and initially exposed to regular medium. The medium was collected at 5 min intervals for 15 min to assess the baseline values of GH and PRL. The pituitaries were then divided into two separate groups; one group received medium containing GHRP-6 and the other group received medium containing PPZ. The samples were collected for 15 min at 5 min intervals after administration of GHRP-6 or PPZ. The levels of GH and PRL were measured using EIA kits. AMNP pituitaries responded positively to different doses of GHRP-6 by secreting high levels of GH at all time points (Figure 6A,C,E,G). In contrast, the parous pituitaries showed a remarkably reduced response at different times and different doses of GHRP-6 (Figure 6B,D,F,H). Next, we measured the levels of PRL secreted by pituitaries in response to PPZ. Similar to the GH results, the baseline levels of PRL were first determined after stabilizing the flow rate of the medium without the secretagogue, and samples were collected at 5 min intervals for 15 min. As expected, different doses of PPZ induced significant responses to PRL secretion from the pituitaries of AMNP animals (Figure 7A,C,E,G). In contrast, the same doses of PPZ were not significantly effective in increasing the secretion of PRL in parous pituitaries (Figure 7B,D,F,H). Together, these results showed that parity induced persistent alterations in the neuroendocrine regulation of the hypothalamic–pituitary axis, causing mammary glands to be refractory to carcinogenesis.

## 4. Discussion

Mammary gland growth in women is influenced by a myriad of hormones throughout various physiological stages of life. The same hormones responsible for the normal growth of the mammary gland can also promote the growth of mammary cancers if not appropriately regulated at each stage of life. Research has been conducted to understand the role of estrogens, and to some extent, the role of progesterone during breast carcinogenesis, which has resulted in current gold standard treatments for estrogen receptor positive breast cancer. Understanding the role of hormones in mammary carcinogenesis is therefore of utmost importance. 

It is well-known that during pregnancy, there are major alterations in levels of several hormones. It is also known that pregnancy early in life reduces a woman’s lifetime risk of breast cancer by almost 50%, when compared to an age-matched nulliparous woman [1,2,3,4,5,6]. There are several theories that have attempted to explain this phenomenon of parity-induced protection against breast cancer [10,11,12,13,14,23,24]. We have previously shown that there is an alteration in systemic levels of hormones in parous animals. In the present study, we showed that GH and PRL were two major hormones that were persistently altered due to parity, which could lead to a decreased risk of mammary carcinogenesis. GH and PRL are known mammogenic hormones that play significant roles in promoting mammary carcinogenesis [25,26,27]. Our results showed that there was a persistent decrease in the tissue and circulating levels of GH and PRL in parous animals, which could play a major role in reducing the risk of breast cancer. 

It is known that circulating levels of GH are higher in about 40% of breast cancer patients [28]. Acromegalics have an increased risk of cancers including breast and colon cancers [29]. Furthermore, no incidence of breast cancer has been observed in patients with Laron syndrome where the GHR is non-functional [30]. GH-deficient and spontaneously dwarf rats are highly resistant to mammary carcinogenesis [31,32,33]. Administration of exogenous GH to dwarf rats exposed to carcinogens results in a high incidence of mammary cancers [32,34]. Furthermore, the mean serum GH levels are significantly decreased in parous women and rats, when compared to their nulliparous counterparts [35]. Our data indicate that parity induces a long-term persistent decline in the levels of GH and also a decreased response of the hypothalamic–pituitary axis to secretagogues. Based on the earlier findings and our current data, we suggest that the persistent decrease in GH could be a reason for parity-induced protection against breast cancer. 

Human GH is also able to bind to the prolactin receptor (PRLr), whose signaling can modulate proliferation, survival, motility, angiogenesis, and differentiation in breast cancer [19,34,36]. Previous studies have shown that parous BALB/c mice are refractory to MNU-induced mammary carcinogenesis and that this refractoriness is not permanent, but can be overcome by hormonal stimulation mediated by pituitary isografts that secrete high levels of PRL [37]. In a pooled analysis of approximately 80% of the world’s prospective data, the relative risk comparing women in the top vs. bottom quartile of prolactin levels was 1.3 (95% confidence interval, p-trend = 0.002). These results were similar for premenopausal and postmenopausal women. Importantly, high prolactin levels were associated with a 60% increased risk of estrogen receptor positive tumor [36]. PRL confers resistance against cisplatin by activating the detoxification enzyme, glutathione-S-transferase, thereby reducing drug entry into the nucleus. These results provide a rational explanation for the ineffectiveness of cisplatin in breast cancer, which is characterized by high expressions of both PRL and its receptor [38]. However, platinum agents like cisplatin and carboplatin as neoadjuvant therapies for triple negative breast cancers have been shown to significantly increase the pathologic complete response [39]. Human mammary epithelial cells harboring degradation-resistant PRLr display accelerated proliferation and increased invasive growth. Conversely, a decrease in PRLr levels achieved by either pharmacological or genetic means in human breast cancer cells dramatically reduces the transformation and tumorigenic properties of these cells [40]. In our investigation, we observed that parity not only led to a decrease in the circulating levels of PRL, but it also persistently reduced the response of hypothalamic–pituitary axis secretagogues. These findings along with previous data indicate that parity reduces the risk of breast cancer by lowering the levels of prolactin and persistently altering the hypothalamic–pituitary axis. The results from the rodent model system are expected to have implications for human breast cancers as well.

## 5. Conclusions

Both GH and PRL are mainly synthesized and secreted by the pituitary under the influence of the hypothalamus. These two hormones have been demonstrated to play a key role in normal breast development as well as in breast cancer development. Our findings here showed that parity affected the hypothalamic–pituitary axis, resulting in long-term decreased levels of GH and PRL. Further, parity also decreased the responsiveness of the hypothalamic–pituitary axis to GH and PRL secretagogues. These findings suggest that parity reduces the life-time risk of breast cancer by altering the neuroendocrine regulation of the hypothalamic–pituitary axis. In conclusion, an effective mechanism for reducing the levels of GH and PRL would therefore provide a powerful tool for decreasing breast cancer risk.

## Figures and Tables

**Table 1:** (**A**). Mammary cancer incidence. (**B**). Mammary cancer multiplicity. (**C**). Mammary cancer latency.

(**A**): Mammary cancer incidence				
**Group/Timepoint**	**18 weeks**	**24 weeks**	**30 weeks**	**36 weeks**
Control	30%	65%	100%	100%
Parous	0%	0%	10%	10%
(**B**): Mammary cancer multiplicity				
**Group/Timepoint**	**18 weeks**	**24 weeks**	**30 weeks**	**36 weeks**
Control	1 tumor/tumor-bearing rat	2.5 tumors/tumor-bearing rat	3.2 tumors/tumor-bearing rat	5.9 tumors/tumor-bearing rat
Parous	0 tumors/tumor-bearing rat	0 tumors/tumor-bearing rat	1 tumor/tumor-bearing rat	1 tumor/tumor-bearing rat
(**C**): Mammary cancer latency				
**Group**		**Days**		
Control		158 ± 32		
Parous		221 ± 25		

## Data Availability

Not applicable.

## Supplementary Materials

The following are available online at https://www.mdpi.com/article/10.3390/cancers13133207/s1, Figure S1: Circulatory levels of growth hormone (GH) in parous and age-matched nulliparous control rats at various timepoints, Figure S2: Circulatory levels of prolactin (PRL) in parous and age-matched nulliparous control rats at various timepoints, Table S1A: Mammary dancer incidence, Table S1B: Mammary cancer multiplicity, Table S1C: Mammary cancer latency.
