# Supplementary material for: Pregnancy Inhibits Mammary Carcinogenesis by Persistently Altering the Hypothalamic–Pituitary Axis
Source: Cancers (Basel). 2021 Jun 26;13(13):3207. doi: 10.3390/cancers13133207 (PMC8267621; doi:10.3390/cancers13133207)
Supplement: Supplementary file 1 [file cancers-13-03207-s001.zip › cancers-1219890-supplementary.pdf]

cancers

Pregnancy inhibits mammary carcinogenesis by persistently altering the hypothalamic-pituitary axis

Ramadevi Subramani[1,2], Adriana Estrada[3], Madeline Dixon[4], Maria Parada[2], Sheryl Rodriguez[1], Diego A. Pedroza[2], Matthew D. Ramirez[4], Alexa Clift[2], Lilia Garcia[2] and Rajkumar Lakshmanaswamy[1,2,*]

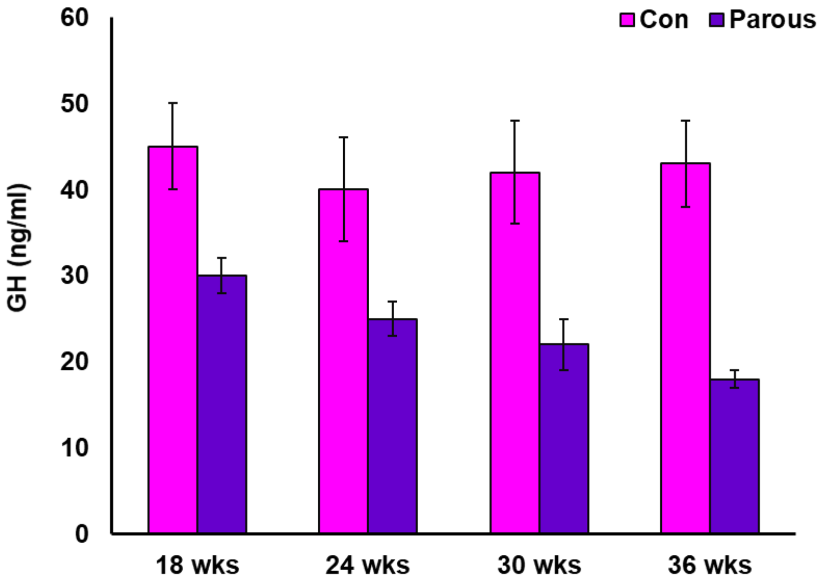

Figure S1. Circulatory levels of growth hormone (GH) in parous and age-matched nulliparous control rats at various timepoints.

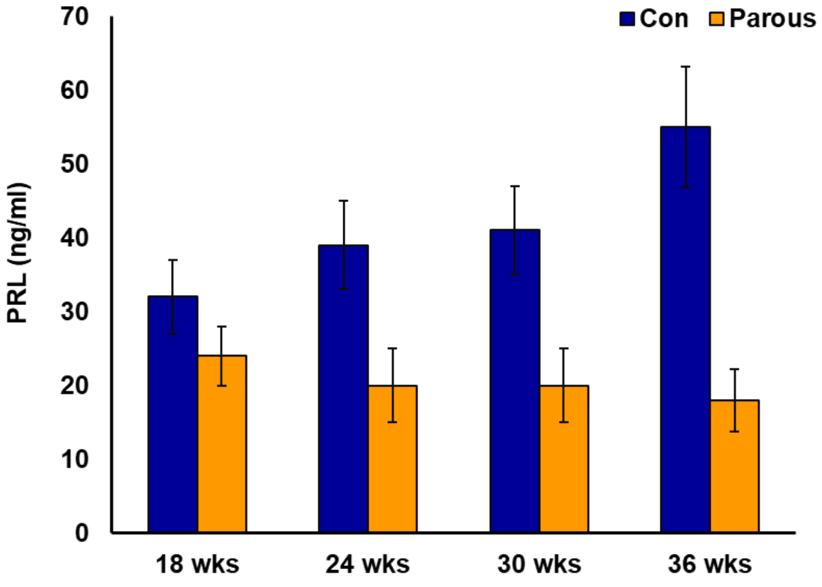

Figure S2: Circulatory levels of prolactin (PRL) in parous and age-matched nulliparous control rats at various timepoints.
